# Burnout and Resilience Following a PERMA‐Informed Happiness Intervention: A Single‐Group Repeated‐Measures Study of EFL Teachers

**DOI:** 10.1002/brb3.71839

**Published:** 2026-10-15

**Authors:** Shuxuan Song, Goudarz Alibakhshi

**Affiliations:** ^1^ College of Arts Yanbian University Yanji China; ^2^ Tarbiat Modares University Tehran Iran

**Keywords:** PERMA, positive psychology, resilience, teacher burnout

## Abstract

**Purpose:**

Teacher burnout is an occupational health concern characterized by physical fatigue, cognitive weariness, and emotional exhaustion. This study examined longitudinal changes in burnout and resilience following a 10‐session online PERMA‐informed happiness intervention among junior high school English as a Foreign Language teachers in Tehran, Iran.

**Method:**

A prospective, uncontrolled single‐group study assessed 70 enrolled EFL teachers; 65 contributed post‐test data, and 61 contributed 6‐month follow‐up data. The 61 complete cases were analyzed with repeated‐measures ANOVA. Summary‐statistic‐based contrasts were calculated under the pooled‐error repeated‐measures model.

**Findings:**

The archived omnibus analyses showed time‐related variation in total burnout (*F*(2, 120) = 12.34, uncorrected *p* < 0.001, *ηp* ^2^ = 0.171) and resilience (*F*(2, 120) = 15.78, uncorrected *p* < 0.001, *ηp* ^2^ = 0.208). Mean burnout was 3.80, 3.05, and 3.15 at baseline, post‐test, and 6 months, respectively; corresponding resilience means were 3.20, 3.95, and 3.90. Under the pooled‐error model, both post‐baseline contrasts differed from baseline (unadjusted *p* < 0.001) for each total score; post‐test and follow‐up did not differ significantly. Original individual‐level data and sphericity diagnostics were unavailable to verify these conditional contrasts.

**Conclusion:**

Burnout and resilience followed distinct within‐group trajectories after participation in the PERMA‐informed program. Model‐based contrasts indicated baseline‐to‐later differences, conditional on the assumed pooled‐error model. The uncontrolled design, baseline‐based selection, missing participant‐level data, and unverified sphericity assumptions preclude a causal interpretation.

## Graphical abstract

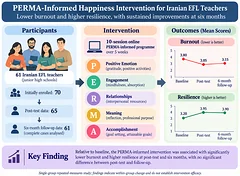

## 1 Introduction

Teacher burnout is a psychologically consequential form of occupational strain that is especially relevant in high‐demand educational settings. Persistent workload, emotional labor, and insufficient organizational resources can contribute to physical fatigue, cognitive weariness, and emotional exhaustion, with consequences for work engagement, job satisfaction, teaching quality, and intentions to leave the profession (Aknin et al. 2011; Diener et al. 2018Hakanen et al. 2006; McInerney et al. 2018; Schaufeli and Bakker 2004). Within EFL contexts, teacher psychological resources such as self‐efficacy have likewise been associated with burnout, psychological well‐being, job satisfaction, and professional functioning (Alibakhshi et al. 2020). In this study, burnout is therefore treated primarily as an occupational mental‐health‐related outcome rather than simply as a professional‐development concern.

Resilience represents a complementary psychological resource because it concerns adaptation and recovery under continuing adversity (Southwick et al. 2014). Teacher resilience has been associated with sustained commitment and functioning despite workload, emotional, and institutional pressures (Beltman et al. 2011). From an occupational perspective, resilience does not remove job demands; rather, it may help explain why individuals differ in their capacity to cope with and recover from those demands(Pressman 2019).

The intervention framework for the present study was Seligman's PERMA model, which conceptualizes well‐being through Positive Emotion, Engagement, Relationships, Meaning, and Accomplishment (Lambert et al. 2015; Seligman 2011; Seligman and Csikszentmihalyi 2000). These domains provide a practical structure for activities intended to cultivate positive affect, absorption in valued activities, interpersonal resources, professional purpose, and attainable goals(Ryan and Deci 2001). PERMA is used here to organize the intervention content rather than to imply that each domain was measured as a mediator.

Broaden‐and‐build theory provides the principal explanatory framework for why positive psychological activities might be associated with later coping resources. The theory proposes that positive affective experiences can broaden momentary thought‐action repertoires and, over time, support enduring psychological and social resources (Fredrickson 2001). In the present study, this framework makes increased resilience and lower burnout scores theoretically plausible after participation, but it does not establish the mechanisms responsible for any observed change because positive affect and other candidate mediators were not directly assessed(Shoman et al. 2023).

Positive psychology interventions commonly include gratitude, mindfulness, reflection, meaning‐focused activities, and goal setting (Layous and Lyubomirsky 2014; Lyubomirsky et al. 2005). Empirical findings across educational and other high‐stress settings are generally favorable but heterogeneous, with results varying by intervention content, comparison condition, implementation quality, and study design (Brassington and Lomas 2021; Sabatini 2014; Shoshani and Steinmetz 2014). Sustainable change may also depend on intentional activity, person‐activity fit, and continued practice (Sheldon and Lyubomirsky 2021). These findings justify further occupationally focused research while underscoring the need to distinguish longitudinal score change from demonstrated intervention efficacy.

A first gap concerns population. Longitudinal positive‐psychology intervention evidence remains limited for EFL teachers, whose work combines pedagogical demands with intensive communication, emotional regulation, classroom interaction, assessment responsibilities, and institutional expectations. These overlapping demands may create a distinctive context for examining burnout‐related strain and resilience resources (Sutton and Wheatley 2003).

A second gap concerns context. Much positive‐psychology intervention research has been conducted in settings that differ from the Iranian educational context. Iranian teachers may work within institutional conditions marked by centralized decision‐making, large classes, and constrained professional autonomy, while meaning, family cohesion, social relationships, and spiritual resources may carry context‐specific significance (Konuralp and Topping 2023; Mahdian Rad and Baleghizadeh 2025). Examining these outcomes in Tehran therefore contributes evidence from a less frequently studied sociocultural and occupational setting without assuming that the same patterns will generalize elsewhere.

A third gap is temporal. Many well‐being interventions emphasize immediate pretest–post‐test change, whereas evidence about whether group‐level score differences remain observable months later is less common. A 6‐month assessment is therefore informative for describing the trajectory from baseline to immediate change and then to longer‐term follow‐up, even though such a trajectory cannot by itself identify the cause of the change.

The present design does not address a causal‐efficacy gap. Because no control or comparison condition was included, maturation, regression to the mean, expectancy effects, repeated‐measures effects, changes in workload, and other time‐varying influences remain possible explanations. The study was accordingly designed and interpreted as a preliminary longitudinal investigation of within‐group trajectories of burnout and resilience following participation in a PERMA‐informed program.

Against this background, the study examined burnout and resilience at baseline, immediately after the 5‐week program, and 6 months later among Iranian junior high school EFL teachers. PERMA served as the intervention framework, broaden‐and‐build theory provided the primary explanatory lens, and occupational stress perspectives were used selectively to situate burnout within the demands and resources of teachers' work.

**Question 1**. How did burnout and its dimensions change from baseline to immediate post‐intervention assessment and 6‐month follow‐up?
**Hypothesis 1**. *Burnout scores would be lower at immediate post‐intervention assessment and 6‐month follow‐up than at baseline*.
**Question 2**. How did resilience and its dimensions change across the same three assessment periods?
**Hypothesis 2**. *Resilience scores would be higher at immediate post‐intervention assessment and 6‐month follow‐up than at baseline*.

## 2 Methods

### 2.1 Research Design

This prospective study used an uncontrolled, single‐group repeated‐measures design with assessments at baseline, immediate post‐intervention, and 6‐month follow‐up. The design permits estimation of within‐participant change over time but does not support causal attribution because no randomized or nonrandomized comparison condition was included. The study planning record documented an a priori G*Power calculation for a medium within‐person effect (*f* = 0.25), three measurements, *α* = 0.05, power = 0.80, and a minimum target of 54 complete participants; 70 teachers were enrolled to allow for attrition. The archived power‐analysis output was not retained, so the assumed correlation among repeated measures and the nonsphericity correction cannot be verified from the available source records.

#### 2.1.1 Sampling and Participant Selection

Of the 300 junior high school EFL teachers initially contacted, 287 completed the SMBM screening (95.7%). Seventy teachers were enrolled after meeting the elevated‐burnout screening rule used during recruitment and agreeing to participate. The retained study documentation does not contain the numerical SMBM cutoff, percentile, or normative criterion applied at screening; therefore, the exact eligibility threshold cannot be reproduced from the archived materials. This limitation is important because selection on elevated baseline burnout can increase susceptibility to regression to the mean. Purposive recruitment and voluntary participation also increase the possibility of self‐selection. Sixty‐five participants completed the immediate post‐intervention assessment, and 61 completed the 6‐month follow‐up, corresponding to retention of 92.9% and 87.1% of the enrolled sample, respectively.

The enrolled sample (*N* = 70) included 47 women (67.1%) and 23 men (32.9%), aged 28–54 years (M = 39.2, SD = 6.7), with a mean of 12.4 years of teaching experience (SD = 4.1). Sixty‐three participants (90.0%) worked full time, and 48 (68.6%) were married. All held at least a bachelor's degree in English or an education‐related field. Demographic data were collected descriptively; no adequately powered moderator analyses were conducted. Participant flow is presented in Figure 1.

**FIGURE 1:**
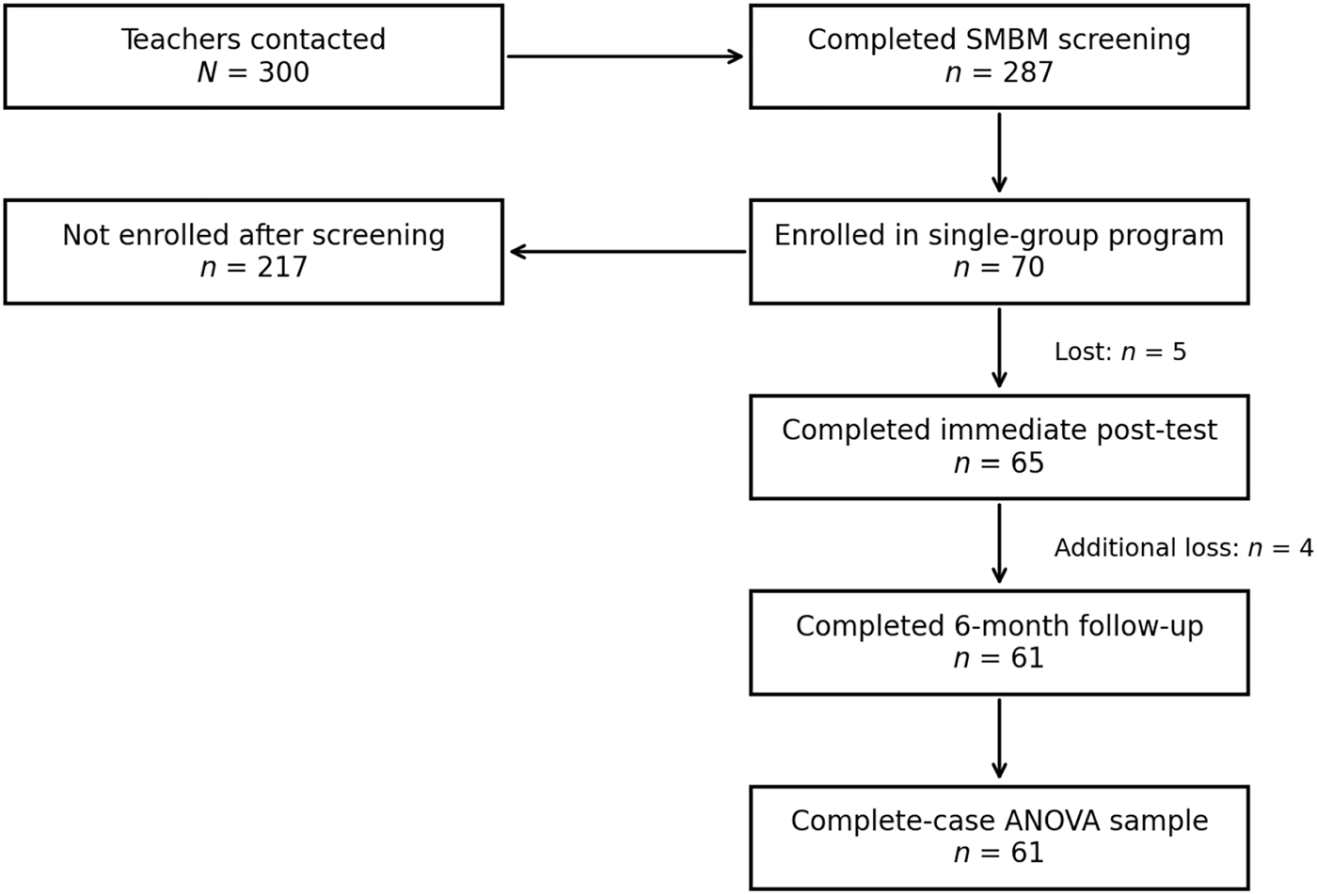
Participant flow through screening, enrollment, post‐test, follow‐up, and complete‐case analysis. Numbers represent participants. The study used a single‐group design with no randomization or control condition; repeated‐measures ANOVA was conducted on *n* = 61 complete cases.

### 2.2 Ethical Considerations

The study was reviewed and approved before recruitment by the Research Ethics Committee of the Research Institute for Culture and Communication, Allameh Tabataba'i University. Under approval/reference number 2024/11/2322. All participants provided written informed consent before participation, and the procedures were conducted in accordance with applicable institutional ethical requirements and the principles of the Declaration of Helsinki.

### 2.3 Instruments

#### 2.3.1 Shirom–Melamed Burnout Measure (SMBM)

The 14‐item SMBM assesses physical fatigue (six items), cognitive weariness (five items), and emotional exhaustion (three items) on a 7‐point frequency scale ranging from 1 (*never*) to 7 (*always*; Shirom and Melamed 2006). Higher item means indicate more frequent burnout symptoms; the measure is dimensional and was not used to make a clinical diagnosis. In the present sample, Cronbach's alpha was 0.84 for the total score, 0.81 for physical fatigue, 0.79 for cognitive weariness, and 0.77 for emotional exhaustion.

#### 2.3.2 Teachers’ Resilience Scale (TRS)

The TRS (Daniilidou and Platsidou 2018) comprises 26 items assessing Personal Competence and Persistence (nine items), Spiritual Influences (three items), Family Cohesion (seven items), and Social Skills and Peer Support (seven items). In the original instrument, the first two subscales use five response categories scored from 0 to 4, whereas the latter two use 5‐point semantic‐differential response formats scored from 1 to 5; higher subscale scores indicate greater resilience resources. The Persian version used in the present study was prepared through forward translation followed by back‐translation. The retained project documentation does not record the translators’ professional qualifications, the reconciliation procedure used to resolve translation discrepancies, formal cultural‐adaptation procedures, pilot testing of the Persian wording, or documentation concerning permission for translation or use. These aspects of the adaptation therefore cannot be independently verified. In the intervention sample, Cronbach's alpha was 0.89 for the total score and ranged from 0.78 to 0.86 across subscales. A four‐factor CFA was conducted in this same intervention sample (*N* = 70); given the sample size relative to the 26‐item model, the CFA results are not used as evidence of independent validation. The present findings should not be interpreted as validating the Persian version of the TRS.

### 2.4 PERMA‐Informed Happiness Intervention

The study used a structured PERMA‐informed happiness intervention organized around Positive Emotion, Engagement, Relationships, Meaning, and Accomplishment (Seligman 2011). The program combined brief instruction with experiential activities addressing positive emotion, engagement, supportive relationships, professional meaning, attainable goals, stress regulation, and self‐care.

Intervention components were distinguished from hypothesized psychological processes and measured outcomes. Gratitude journaling and mindfulness were program activities; positive affect, emotion regulation, meaning, self‐efficacy, and social connectedness were plausible processes but were not measured as mediators. The only longitudinal outcomes analyzed were burnout and resilience. Accordingly, the study does not attribute observed score trajectories to any specific component or mechanism.

The program comprised 10 sequential 90‐min online sessions delivered twice weekly over 5 weeks by one licensed counseling psychologist. Activities included reflective practice, group discussion, mindfulness exercises, gratitude journaling, stress‐management practice, and personal goal setting. Table 1 presents the planned content and activities.

**TABLE 1:** Session content and planned activities for the 10‐session PERMA‐informed program.

Session	Topic	Objective	Content	Activities
1	Introduction to Positive Psychology and Happiness	Introduce the fundamental concepts of positive psychology and happiness training.	Overview of positive psychology, Seligman's PERMA model, and the role of happiness in teaching and reducing burnout. Pre‐training reflection on happiness levels and emotional exhaustion.	Group discussion on teaching challenges and burnout; sharing personal experiences.
2	Cultivating Positive Emotions	Help teachers enhance positive emotions to counteract burnout.	Techniques for cultivating gratitude and optimism, including mindfulness practices and focusing on positive daily events.	Gratitude journaling (daily three blessings); guided mindfulness meditation.
3	Engagement and Flow in Teaching	Foster engagement and flow in professional activities.	Understanding “flow”; identifying teaching tasks that promote flow; strategies to enhance classroom engagement.	Reflection activity on satisfying tasks; group discussion on achieving flow in teaching.
4	Building Positive Relationships	Improve interpersonal relationships in the workplace.	Emphasizing empathy, communication, and building connections with students, colleagues, and parents.	Role‐playing exercises for handling difficult conversations effectively.
5	Finding Meaning in Teaching	Reconnect teachers with the deeper meaning of their profession.	Exploring the personal and professional significance of teaching, finding purpose in daily tasks, and linking meaning to resilience.	Reflective writing on career motivations; group sharing of personal teaching journeys.
6	Accomplishment and Achievement	Enhance personal and professional achievement.	Setting and celebrating achievable goals; understanding self‐efficacy; tracking progress effectively.	Goal‐setting workshops for short‐ and long‐term objectives.
7	Resilience Building	Develop strategies for emotional and mental resilience.	Understanding resilience, stress management techniques, emotional regulation, and self‐care strategies.	Practicing stress‐relief techniques such as breathing exercises.
8	Mindfulness and Stress Reduction	Introduce mindfulness as a stress‐reduction tool.	Exploring mindfulness for emotional regulation and stress management, integrating it into daily routines.	Guided mindfulness exercises: practice mindful listening and breathing.
9	Sustaining Happiness in the Long‐Term	Equip teachers with strategies for maintaining happiness.	Reviewing previous strategies, incorporating happiness practices into daily teaching, and addressing barriers to sustained happiness.	Developing personal happiness action plans for classroom and professional life.
10	Reflection and Future Planning	Consolidate learning and plan for sustained growth.	Recap of workshop concepts; reflections on emotional well‐being and teaching effectiveness; long‐term planning.	Final group discussion on individual action plans and post‐workshop reflections on happiness and burnout.

*Note*: The program was planned for online delivery twice weekly for 5 weeks; each session was approximately 90 min and was led by one licensed counseling psychologist. The table describes the planned curriculum. No independent fidelity audit, retained attendance‐dose dataset, or structured homework‐adherence measure was available; therefore, session‐level exposure and adherence could not be quantified.

### 2.5 Intervention Fidelity

The intervention was planned around a common written session plan and was delivered by the same facilitator. The retained materials document the planned curriculum and facilitator assignment but do not provide session‐level evidence sufficient to quantify the dose received by individual participants or to verify consistency of delivery across all sessions. No independent fidelity checklist, facilitator‐adherence rating, session‐recording review, or structured supervision rating was retained. Session‐by‐session attendance and homework‐completion records were also unavailable in the analytic archive. Formal feasibility indicators, acceptability measures, implementation outcomes, and prospectively defined fidelity thresholds were not assessed; accordingly, feasibility is not treated as an outcome of the present study.

### 2.6 Procedure

The study was conducted in Tehran, Iran, from January to February 2023, with a 6‐month follow‐up. Approximately 1 week before the first session, participants completed the SMBM and TRS through Google Forms to establish baseline scores.

Participants were purposively recruited from four public high schools and two private institutions across different districts of Tehran, in collaboration with school principals and district education offices. This urban, network‐based recruitment strategy improved feasibility but may have favored teachers who were more connected to professional‐development opportunities and more motivated to participate.

Following this, the intervention phase involved a structured happiness training program delivered over 5 weeks, consisting of 10 sessions held twice weekly, each lasting 90 min. These sessions were conducted online via the Skyroom video conferencing platform, thus ensuring accessibility for all participants. The training followed a standardized curriculum grounded in the PERMA model of well‐being. It included instructional techniques such as short lectures, guided mindfulness practices, group discussions, reflective journaling, and goal‐setting exercises. The sessions were facilitated by a licensed counseling psychologist with documented expertise in implementing positive psychology interventions in educational settings. While the doctoral researcher in education coordinated the overall project design, ethical compliance, and evaluation, the psychologist was solely responsible for delivering the psychological content and interacting with participants during the sessions.

Moreover, active participant engagement was fostered through breakout discussions, peer‐sharing activities, and asynchronous reflection prompts disseminated via WhatsApp, thereby enhancing the collaborative and reflective nature of the experience.

Participants completed the SMBM and TRS immediately after the final session and again 6 months later. Reminder messages and certificates of participation were used to support follow‐up retention.

Of the 70 enrolled participants, 65 completed the post‐intervention assessment; five were unavailable because of scheduling conflicts during examination weeks. Sixty‐one completed the 6‐month follow‐up; the four additional losses comprised two participants on medical leave, one who changed schools, and one who did not respond after repeated contact attempts. Participant‐level baseline data for completer‐versus‐non‐completer comparisons were not available in the retained analytic materials, so no claim of baseline equivalence between attrition groups is made.

### 2.7 Data Analysis

Analyses were conducted in SPSS 26. Descriptive statistics used all observations available at each assessment. The archived primary analysis was a one‐factor repeated‐measures ANOVA based on the 61 participants with complete data at all three assessments; nine enrolled participants were excluded listwise and no values were imputed. For the pairwise comparisons, model‐based mean contrasts, standard errors, *t*‐statistics, and 95% confidence intervals were calculated from the reported complete‐case means and the pooled within‐subject error implied by each retained omnibus *F*‐statistic. These calculations use the uncorrected repeated‐measures model, df = 120, and unadjusted two‐sided *p*‐values. As the original participant‐level covariance data and independently verified SPSS pairwise output were not retained, these contrasts are conditional reconstructions rather than independently validated participant‐level paired comparisons. The two baseline contrasts per outcome remained below 0.001 even under a three‐comparison Bonferroni threshold, but the tabled *p*‐values and confidence intervals are unadjusted. Mixed effects or attrition sensitivity analyses could not be undertaken without the participant‐level data.

The analysis plan called for checking distributional assumptions and Mauchly's test of sphericity and applying Greenhouse–Geisser correction when indicated. Exact Shapiro–Wilk statistics, Mauchly W and χ^2^, Greenhouse–Geisser epsilon, and a correction indicator were not present in the retained output. Accordingly, omnibus *p*‐values and the model‐based contrasts are reported conditional on the archived uncorrected degrees of freedom. As a sensitivity calculation for the omnibus tests, substituting the most conservative lower‐bound epsilon of 0.50 (df = 1, 60) while holding the archived *F*‐values constant yielded *p* < 0.002 for every burnout and resilience outcome. This calculation does not replace the missing original diagnostics and does not validate the pooled‐error pairwise standard errors.

## 3 Results

### 3.1 Participant Demographics

Table 2 summarizes the enrolled sample (*N* = 70). Sixty‐five participants contributed post‐test data, and 61 contributed follow‐up data. The complete‐case inferential sample was therefore *n* = 61, while the baseline demographic description retained all 70 enrolled participants.

**TABLE 2:** Demographic characteristics of the *enrolled* participants (*N* = 70).

Variable	Category	*n* (%)
Gender	Female	47 (67.1)
	Male	23 (32.9)
Age, years	M = 39.2, SD = 6.7	
Teaching *experience*, years	M = 12.4, SD = 4.1	
Employment *status*	Full‐time	63 (90.0)
	Part‐time	7 (10.0)
Marital *status*	Married	48 (68.6)
	Single	10 (14*.3*)
	*Other*	12 (17.*1*)

*Note*: Values are based on all 70 enrolled participants. Percentages may not total exactly 100 because of rounding.

### 3.2 Participant Retention and Missing‐Data Handling

Table 3 distinguishes the enrolled, available‐case, and complete‐case samples. The repeated‐measures analysis used the 61 participants with complete data at all three assessments; nine enrolled participants were excluded listwise, and no imputation was used. Because participant‐level baseline data for those lost to follow‐up were not retained in the available analytic materials, attrition‐group equivalence was not assumed.

**TABLE 3:** Participant retention and analysis populations.

Stage	*n*	% of enrolled (*N* = 70)	Use in analysis
Pretest/enrolled	70	100.0	Baseline description
Immediate post‐test	65	92.9	Available‐case descriptives
Six‐month follow‐up	61	87.1	Available‐case descriptives
Complete data at all time points	61	87.1	Primary repeated‐measures ANOVA; listwise exclusion of 9; no imputation

*Note*: Descriptive summaries used observations available at each time point. The repeated‐measures ANOVA used 61 *complete cases; nine participants were excluded listwise, and no imputation was performed*.

### 3.3 Descriptive Statistics

Table 4 presents complete‐case means, standard deviations, and 95% confidence intervals across the three assessments. Burnout scores decreased from baseline to immediate post‐test, followed by a modest descriptive increase at 6‐month follow‐up; the follow‐up mean remained below baseline. Resilience increased from baseline to post‐test and was similar at 6 months. Pairwise contrasts estimated from the reported pooled‐error ANOVA summaries are presented in Tables 6 and 8.

**TABLE 4:** *Complete‐case* descriptive statistics across *assessment points* (*n* = 61).

Measure	Pretest M (SD) [95% CI]	Post‐test M (SD) [95% CI]	Follow‐up M (SD) [95% CI]
Burnout (SMBM *total*)	3.80 (0.70) *[3.62, 3.98]*	3.05 (0.65) *[2.88, 3.22]*	3.15 (0.60) *[3.00, 3.30]*
Physical *fatigue*	3.75 (0.72) *[3.57, 3.93]*	3.00 (0.68) *[2.83, 3.17]*	3.08 (0.66) *[2.91, 3.25]*
Cognitive *weariness*	3.90 (0.68) *[3.73, 4.07]*	3.20 (0.60) *[3.05, 3.35]*	3.30 (0.58) *[3.15, 3.45]*
Emotional *exhaustion*	3.70 (0.60) *[3.55, 3.85]*	3.00 (0.55) *[2.86, 3.14]*	3.10 (0.52) *[2.97, 3.23]*
Resilience (TRS *total*)	3.20 (0.65) *[3.03, 3.37]*	3.95 (0.60) *[3.80, 4.10]*	3.90 (0.58) *[3.75, 4.05]*
Personal *competence*	3.15 (0.63) *[2.99, 3.31]*	3.90 (0.58) *[3.75, 4.05]*	3.85 (0.55) *[3.71, 3.99]*
Spiritual influences	3.10 (0.68) [2.93, 3.27]	3.85 (0.65) [3.68, 4.02]	3.80 (0.62) [3.64, 3.96]
Family cohesion	3.25 (0.60) [3.10, 3.40]	4.00 (0.55) [3.86, 4.14]	3.95 (0.52) [3.82, 4.08]
Social skills and peer support	3.30 (0.65) [3.13, 3.47]	3.98 (0.60) [3.83, 4.13]	3.92 (0.58) [3.77, 4.07]

*Note*: Values are M (SD) [95% CI] for the *n* = 61 complete‐case sample. SMBM items are rated from 1 to 7, with higher scores indicating more burnout symptoms. Higher TRS scores indicate greater resilience resources.

### 3.4 Repeated‐Measures Results for Burnout

As shown in Table 5, the archived repeated‐measures ANOVAs indicated time‐related variation in total burnout and its subscales (all uncorrected *p* < 0.001). Partial eta squared ranged from 0.153 to 0.191; cognitive weariness had the largest association (*ηp* ^2^ = 0.191). Model‐based within‐group contrasts are presented in Table 6.

**TABLE 5:** Repeated‐measures ANOVA for burnout outcomes (*n* = 61).

Measure	*F*(2, 120)	*p*	*ηp* ^2^	Interpretation
SMBM total	12.34	< 0.001	0.171	Large within‐group time association
Physical fatigue	10.87	< 0.001	0.153	Large within‐group time association
Cognitive weariness	14.21	< 0.001	0.191	Large within‐group time association
Emotional exhaustion	11.45	< 0.001	0.160	Large within‐group time association

*Note*: The *F*‐statistics and degrees of freedom were retained from the original complete‐case analysis (*n* = 61). The reported *p*‐values use uncorrected *F*(2, 120) degrees of freedom because the original Mauchly statistics, Greenhouse–Geisser epsilon values, and the correction indicator were unavailable. A lower‐bound sensitivity calculation using df = (1, 60) produced *p* < 0.002 for every burnout outcome. Partial eta squared describes the association with time, not a treatment‐versus‐control effect.

**TABLE 6:** Pairwise comparisons for burnout outcomes (*n* = 61).

Measure	Comparison	Mean difference	*t*(120)	*p*	95% CI
SMBM total	Pretest–posttest	0.75	4.57	< 0.001	[0.43, 1.07]
	Pretest–follow‐up	0.65	3.96	< 0.001	[0.33, 0.97]
	Follow‐up–post‐test	0.10	0.61	0.543	[−0.22, 0.42]
Physical fatigue	Pretest–posttest	0.75	4.25	< 0.001	[0.40, 1.10]
	Pretest–follow‐up	0.67	3.79	< 0.001	[0.32, 1.02]
	Follow‐up–post‐test	0.08	0.45	0.651	[−0.27, 0.43]
Cognitive weariness	Pretest–posttest	0.70	4.93	< 0.001	[0.42, 0.98]
	Pretest–follow‐up	0.60	4.22	< 0.001	[0.32, 0.88]
	Follow‐up–post‐test	0.10	0.70	0.483	[−0.18, 0.38]
Emotional exhaustion	Pretest–posttest	0.70	4.42	< 0.001	[0.39, 1.01]
	Pretest–follow‐up	0.60	3.79	< 0.001	[0.29, 0.91]
	Follow‐up–post‐test	0.10	0.63	0.529	[−0.21, 0.41]

*Note*: MD = mean difference; CI = confidence interval. Estimates are unadjusted model‐based contrasts calculated from the reported complete‐case means and pooled within‐subject error implied by the archived *F*‐statistics (df = 120), assuming sphericity. They are not independently verified original SPSS pairwise output. Positive pretest‐minus‐later differences indicate lower burnout at the later assessment.

### 3.5 Pairwise Comparisons for Burnout

Under the pooled‐error repeated‐measures model, total burnout was lower at post‐test than baseline (MD = 0.75, 95% CI [0.43, 1.07], *t*(120) = 4.57, unadjusted *p* < 0.001) and lower at 6 months than baseline (MD = 0.65, 95% CI [0.33, 0.97], *t*(120) = 3.96, unadjusted *p* < 0.001). The small follow‐up increase relative to post‐test was not statistically significant (MD = 0.10, 95% CI [−0.22, 0.42], *t*(120) = 0.61, *p* = 0.543). The same direction of change was observed for physical fatigue, cognitive weariness, and emotional exhaustion; both baseline contrasts had unadjusted *p* < 0.001, whereas post‐test‐to‐follow‐up contrasts were not significant (Table 6). These inferences are conditional on the pooled‐error model because the original paired‐observation covariance data were unavailable.

### 3.6 ANOVA Results for Resilience

Table 7 presents the archived repeated‐measures ANOVA results for total resilience and all four subscales (all uncorrected *p* < 0.001). Partial eta squared ranged from 0.177 to 0.208; the largest time association was observed for total resilience (*ηp* ^2^ = 0.208). Model‐based within‐group contrasts are shown in Table 8.

**TABLE 7:** Repeated‐measures ANOVA for resilience outcomes (*n* = 61).

Measure	F(2, 120)	*p*	*ηp* ^2^	Interpretation
TRS total	15.78	< 0.001	0.208	Large within‐group time association
Personal competence	14.56	< 0.001	0.195	Large within‐group time association
Spiritual influences	12.90	< 0.001	0.177	Large within‐group time association
Family cohesion	13.45	< 0.001	0.183	Large within‐group time association
Social skills and peer support	14.23	< 0.001	0.192	Large within‐group time association

*Note*: The *F*‐statistics and degrees of freedom were retained from the original complete‐case analysis (*n* = 61). The reported *p*‐values use uncorrected *F*(2, 120) degrees of freedom because original Mauchly statistics, Greenhouse–Geisser epsilon values, and the correction indicator were unavailable. A lower‐bound sensitivity calculation using df = (1, 60) produced *p* < 0.001 for all five resilience outcomes. Partial eta squared describes the association with time, not a treatment‐versus‐control effect.

**TABLE 8:** Pairwise comparisons for resilience outcomes (*n* = 61).

Measure	Comparison	Mean difference	*t*(120)	*p*	95% CI
TRS total	Post‐test–pretest	0.75	5.02	< 0.001	[0.45, 1.05]
	Follow‐up–pretest	0.70	4.69	< 0.001	[0.40, 1.00]
	Post‐test–follow‐up	0.05	0.33	0.738	[−0.25, 0.35]
Personal competence	Post‐test–pretest	0.75	4.83	< 0.001	[0.44, 1.06]
	Follow‐up–pretest	0.70	4.50	< 0.001	[0.39, 1.01]
	Post‐test–follow‐up	0.05	0.32	0.748	[−0.26, 0.36]
Spiritual influences	Post‐test–pretest	0.75	4.54	< 0.001	[0.42, 1.08]
	Follow‐up–pretest	0.70	4.24	< 0.001	[0.37, 1.03]
	Post‐test–follow‐up	0.05	0.30	0.763	[−0.28, 0.38]
Family cohesion	Post‐test–pretest	0.75	4.64	< 0.001	[0.43, 1.07]
	Follow‐up–pretest	0.70	4.33	< 0.001	[0.38, 1.02]
	Post‐test–follow‐up	0.05	0.31	0.758	[−0.27, 0.37]
Social skills and peer support	Post‐test–pretest	0.68	4.82	< 0.001	[0.40, 0.96]
	Follow‐up–pretest	0.62	4.39	< 0.001	[0.34, 0.90]
	Post‐test–follow‐up	0.06	0.43	0.672	[−0.22, 0.34]

*Note*: MD = mean difference; CI = confidence interval. Estimates are unadjusted model‐based contrasts calculated from the reported complete‐case means and pooled within‐subject error implied by the archived *F*‐statistics (df = 120), assuming sphericity. They are not independently verified original SPSS pairwise output. Positive differences indicate higher resilience at the first‐named assessment.

### 3.7 Pairwise Comparisons for Resilience

Under the pooled‐error repeated‐measures model, total resilience was higher at post‐test than baseline (MD = 0.75, 95% CI [0.45, 1.05], *t*(120) = 5.02, unadjusted *p* < 0.001) and at 6 months than baseline (MD = 0.70, 95% CI [0.40, 1.00], *t*(120) = 4.69, unadjusted *p* < 0.001). The post‐test‐to‐follow‐up contrast was not significant (MD = 0.05, 95% CI [−0.25, 0.35], *t*(120) = 0.33, *p* = 0.738). Comparable model‐based contrasts were found for personal competence, spiritual influences, family cohesion, and social skills and peer support: Both post‐baseline assessments had higher mean scores than baseline (unadjusted *p* < 0.001), but the later assessments did not differ significantly from one another (Table 8). These contrasts depend on the pooled‐error assumption and could not be checked against participant‐level paired data.

Figures 2 and 3 display the mean burnout and resilience subscale trajectories with 95% confidence intervals for the *n* = 61 complete‐case sample.

**FIGURE 2:**
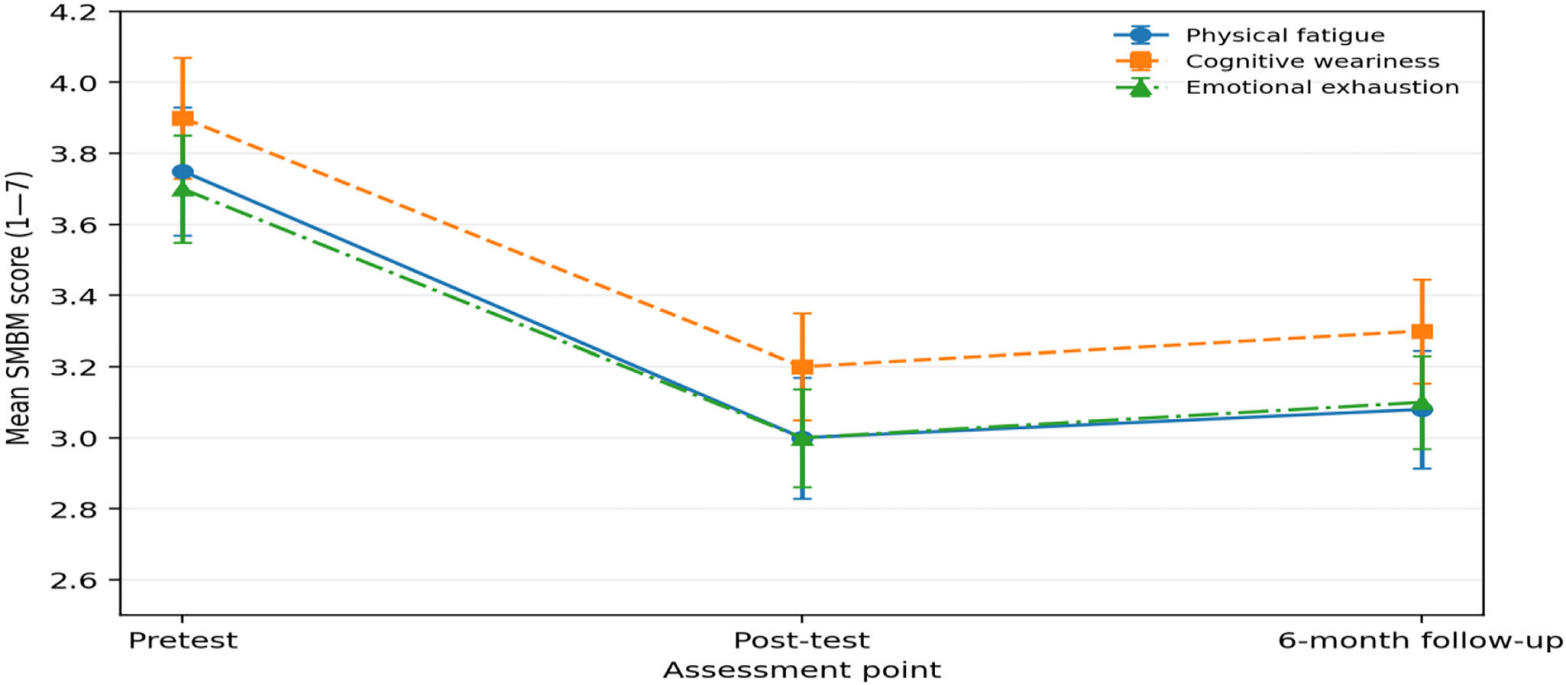
Mean SMBM subscale scores at baseline, immediate post‐test, and 6‐month follow‐up. Error bars are 95% confidence intervals for the 61 complete cases. All three subscales showed lower post‐test and follow‐up means than baseline. Model‐based contrasts were significant for the two baseline comparisons under the assumed pooled‐error model (Table 6).

**FIGURE 3:**
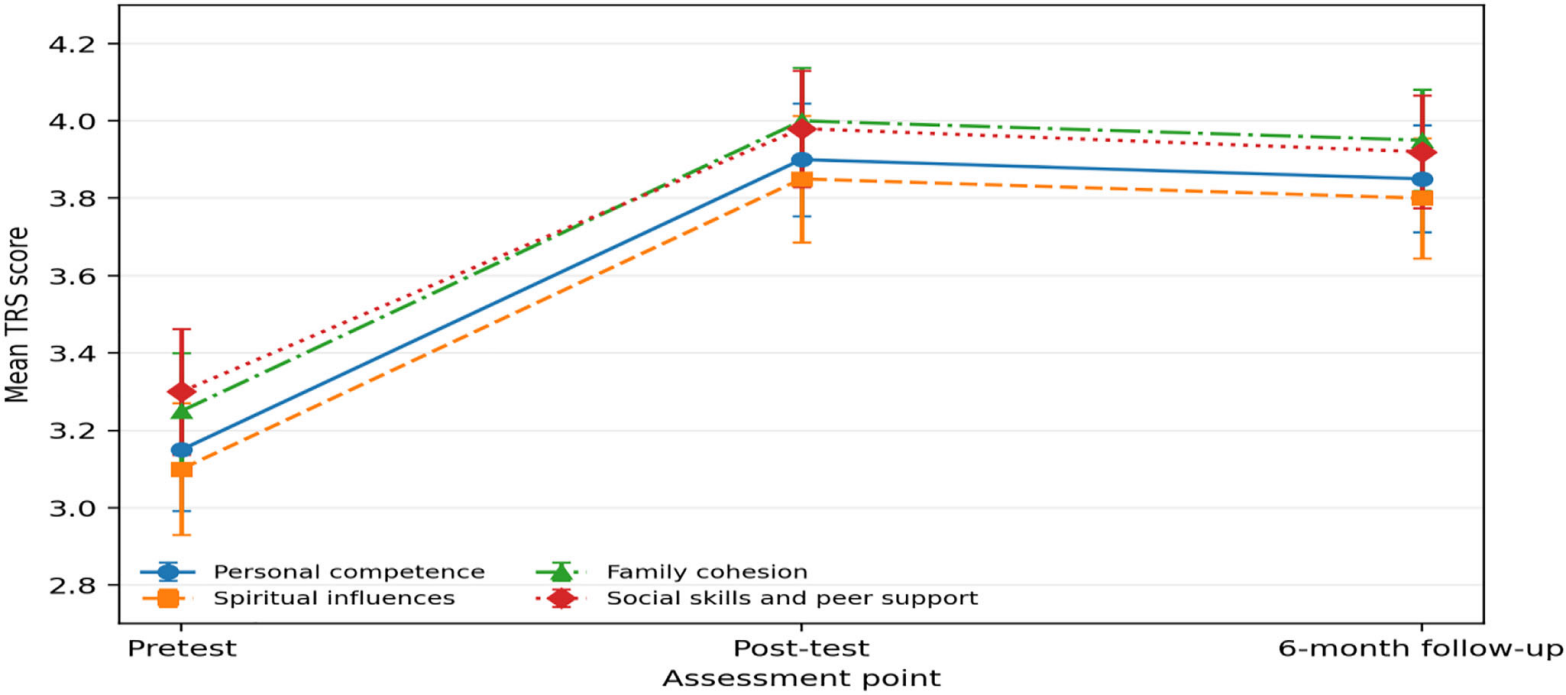
Mean Teachers’ Resilience Scale subscale scores at baseline, immediate post‐test, and 6‐month follow‐up. Error bars are 95% confidence intervals for the 61 complete cases. All four subscales showed higher post‐test and follow‐up means than baseline. Model‐based contrasts were significant for the two baseline comparisons under the assumed pooled‐error model (Table 8).

## 4 Discussion

Burnout and resilience showed different within‐group trajectories after participation: Burnout decreased immediately and increased slightly by 6 months, whereas resilience increased immediately and was close to its post‐test level at follow‐up. Conditional model‐based contrasts suggested that burnout was lower and resilience higher at both later assessments than at baseline, without significant differences between post‐test and follow‐up. The results should not be taken as evidence that the program caused these changes; no comparison group was included, participants were selected for elevated burnout, and the paired‐data assumptions underlying the reconstructed contrasts could not be checked.

PERMA and broaden‐and‐build theory offer a coherent psychological interpretation of these trajectories. Positive‐emotion activities may have broadened coping repertoires; relationship‐focused exercises may have strengthened perceived interpersonal resources; meaning‐oriented reflection may have reinforced professional purpose; and goal setting may have supported competence‐related perceptions (Camacho‐Morles et al. 2021; Fredrickson 2001; Layous and Lyubomirsky 2014). Mindfulness and stress‐regulation activities may also have supported emotion regulation. These are candidate mechanisms only: The study measured burnout and resilience, not positive affect, mindfulness, meaning, self‐efficacy, social connectedness, or mediation pathways.

The study also contributes evidence from an occupational and sociocultural context that is less represented in positive‐psychology intervention research. EFL teaching combines communicative, emotional, pedagogical, and institutional demands, while the Iranian setting may shape how professional meaning, family cohesion, peer support, and spiritual resources are experienced. Related evidence from Iranian EFL teachers has shown that teacher self‐efficacy is interconnected with burnout, psychological well‐being, engagement, job satisfaction, emotion regulation, and professional development, further illustrating the interdependence of personal and occupational resources in this context (Liu and Xie 2025; Mossafaie et al. 2024; Wang et al. 2024). The resilience subdomains therefore provide useful descriptive information beyond a global score, although their cultural interpretation requires cross‐context replication rather than assuming universality (Cohen et al. 2023; Southwick et al. 2014).

Total burnout was 0.75 points lower immediately after the program and 0.65 points lower at 6 months than at baseline; total resilience was 0.75 and 0.70 points higher at these assessments. The model‐based baseline contrasts were statistically significant under the pooled‐error assumption, but neither instrument was used diagnostically, and no minimally important difference or clinical recovery threshold was prespecified. These results describe score changes rather than remission or demonstrated clinical improvement.

Practical interpretation should remain occupational rather than purely individual. A PERMA‐informed program may be considered one supportive component within a broader teacher well‐being strategy, but it should not replace organizational action on workload, professional autonomy, administrative support, class size, employment security, and access to mental‐health resources. Individual resilience resources and structural working conditions should be addressed together.

Several limitations constrain inference. With no comparison condition and selection based on elevated baseline burnout, regression to the mean, expectancy, repeated measurement, seasonal workload, and other time‐varying factors remain alternative explanations. The numerical SMBM recruitment cutoff could not be recovered, limiting reproducibility. Complete‐case ANOVA excluded nine enrolled participants, and the original participant‐level longitudinal data were unavailable for mixed‐effects models, attrition analyses, or verification of within‐person pairwise variability. Sphericity diagnostics and the archived correction indicator were also unavailable. Although the reported *F*‐values remained significant in a lower‐bound degrees‐of‐freedom sensitivity calculation, the pairwise *t*‐statistics and confidence intervals were reconstructed from aggregate data using an assumed pooled error term; these inferential comparisons require confirmation against the original analysis output or participant‐level data. The pairwise *p*‐values were unadjusted across outcomes. Session‐level attendance, homework completion, independent fidelity, formal feasibility outcomes, and psychological mediators were not measured systematically. Documentation concerning the Persian TRS translation and cultural adaptation was incomplete, and the present sample does not independently validate that version. Finally, the Tehran EFL‐teacher sample limits generalizability to other educational and occupational contexts.

The next step is a fully powered randomized study with an active attention‐matched comparison condition, prospectively specified fidelity and adherence measures, participant‐level mixed‐effects analysis, and longer follow‐up. Direct measurement of positive affect, mindfulness, emotion regulation, meaning, self‐efficacy, and perceived social support would allow candidate mechanisms to be tested rather than inferred. Broader sampling and cross‐cultural measurement work would clarify whether the observed trajectories replicate beyond the present occupational and cultural setting.

### 4.1 Implications and Future Research

For practice, the program can be considered a structured, low‐intensity occupational well‐being resource that warrants more rigorous prospective evaluation. The present study did not formally assess feasibility, acceptability, implementation outcomes, session‐level dose, or fidelity. Future implementation studies should therefore use a written facilitator manual, prospective attendance records, homework or practice logs, acceptability measures, and independent fidelity checks so that exposure, adherence, and implementation quality can be quantified.

For institutions, individual well‐being programs should complement rather than substitute for organizational prevention. Workload allocation, professional autonomy, administrative support, class size, job security, and access to psychological services remain important determinants of occupational strain. A credible teacher well‐being strategy should therefore combine resource‐building opportunities with efforts to reduce modifiable job demands.

For research, randomized controlled designs with active comparison conditions should be paired with linear mixed‐effects models that use all available repeated observations and with prespecified missing‐data sensitivity analyses. Future studies should define meaningful‐change criteria in advance, measure hypothesized psychological processes directly, monitor intervention dose and fidelity, and recruit across disciplines, rural and urban settings, Iranian regions, and other countries to evaluate generalizability and cultural adaptation.

## 5 Conclusion

In this uncontrolled single‐group study, mean burnout declined and mean resilience increased after participation in a 10‐session PERMA‐informed program. Model‐based comparisons calculated from the retained summary statistics indicated differences between baseline and both post‐baseline assessments under the pooled‐error repeated‐measures model, with no significant contrast between post‐test and 6‐month follow‐up. Missing original sphericity diagnostics and participant‐level data limit verification of these contrasts, and the findings do not establish intervention efficacy.

A randomized controlled study with an active comparison condition, participant‐level mixed‐effects analysis, prospectively monitored fidelity and adherence, and direct assessment of candidate mechanisms is needed to determine whether the program itself produces durable reductions in burnout and increases in resilience.

## Author Contributions

**Shuxuan Song**: conceptualization, writing – original draft, writing – review and editing. **Goudarz Alibakhshi**: conceptualization, writing – original draft, methodology, writing – review and editing, supervision.

## Funding

The authors have nothing to report.

## Ethics Statement

The study was reviewed and approved before recruitment by the the Research Institute for Culture and Communication, Allameh Tabataba'i University. Under approval/reference number 2024/11/2322. All participants provided written informed consent before participation, and the procedures were conducted in accordance with applicable institutional ethical requirements and the principles of the Declaration of Helsinki.

## Conflicts of Interest

The authors declare no conflicts of interest.

## Data Availability

The study data have not been deposited publicly. The participant‐level longitudinal dataset was unavailable to the authors during preparation of this manuscript; its current custodian and retention status could not be established from the available project documentation. Retained analytical materials comprised the reported aggregate descriptive summaries and omnibus ANOVA statistics. The pairwise contrasts presented here were calculated from those aggregate results under the pooled‐error repeated‐measures model and could not be independently verified using participant‐level observations. Thus, de‐identified participant‐level data cannot currently be offered for sharing. The available documentation did not establish an institutional retention record specific to these data.
